# The phase angle cut-off point capable of discriminating hemodialysis patients with reduced exercise tolerance: a cross-sectional study

**DOI:** 10.1186/s13102-024-00825-5

**Published:** 2024-02-02

**Authors:** Davi de Souza Francisco, Igor Gutierrez Moraes, Camila Porto Brito, Renato Fraga Righetti, Wellington Pereira Yamaguti

**Affiliations:** Hospital Sírio-Libanês, Rehabilitation Service, São Paulo, São Paulo Brazil

**Keywords:** Chronic kidney disease, Electric impedance, Phase angle, Exercise test, Functional capacity, Step test

## Abstract

**Background:**

Phase angle (PhA) is a prognostic marker of all-cause mortality in chronic kidney disease. However, no study has investigated this marker as a predictor of exercise intolerance in hemodialysis (HD) patients. The aim of this study was to determine a cut-off point for the PhA capable of discriminating HD patients with reduced exercise tolerance.

**Methods:**

Thirty-one patients (80.6% men, median age 69 years) were included. The evaluations were performed on three different days, before the HD session. The outcomes evaluated were: biochemical markers, inflammatory and nutritional status, body composition, peripheral muscle strength and exercise tolerance. Performance ≤50% of the predicted value in the six-minute step test (6MST) was defined as reduced exercise tolerance.

**Results:**

Patients presented an average of 67.6 steps (50.5% of predicted) in the 6MST. Fifteen patients (48.4%) were classified with reduced exercise tolerance. The receiver operating characteristic curve indicated a cut-off point of 3.73° for the PhA (sensitivity = 87%, specificity = 81%, and area under the curve = 0.88 [95% CI: 0.76–1.00]; *p* < 0.001). Patients with reduced exercise tolerance had worse inflammatory and nutritional status, lower PhA and greater impairment of peripheral muscle strength.

**Conclusion:**

The cut-off point of 3.73° for the PhA is sensitive and specific to discriminate HD patients with reduced exercise tolerance.

**Trial registration:**

This study was registered in the Clinical Trials database (no. NCT03779126, date of first registration 19/12/2018).

**Supplementary Information:**

The online version contains supplementary material available at 10.1186/s13102-024-00825-5.

## Background

Chronic kidney disease (CKD) causes changes in the muscular, cardiovascular and respiratory systems [1–5]. Consequently, the non-harmonious functioning between these systems can generate exercise intolerance and favor the emergence of sedentary behavior in this population [6]. Furthermore, in patients with CKD, lower levels of physical activity were associated with mortality [7–9]. For that reason, it is extremely important to monitor the exercise tolerance in these individuals. Nevertheless, functional assessment is not a reality in many nephrology centers [10]. Thus, it is necessary to investigate whether evaluation markers already used in this population could facilitate the screening and referral of patients to inter or intradialytic rehabilitation programs.

Bioelectrical impedance analysis (BIA) is a tool widely used in hemodialysis (HD) patients, as it allows for the assessment of body composition, monitoring of nutritional status, better fluid management, and estimation of dry weight [11, 12]. In addition, by means BIA, it is possible to measure the phase angle (PhA), a variable that reflects the integrity of the cell membrane, the number of cells, and the performance of their functions [11, 13, 14]. In patients with CKD, lower PhA values ​​were associated with unfavorable clinical outcomes, such as protein energy-wasting, frailty, infection, cardiovascular and all-cause mortality [15–18]. Furthermore, Brito et al. [19] observed a high correlation between PhA and exercise tolerance, another variable associated with mortality, in a sample of HD patients.

Given the above, PhA seems to be a marker that could be used by different health team members (dieticians, nurses, and physicians) to screen HD patients who should start a rehabilitation program. However, no study has investigated whether this marker can predict exercise intolerance in this population. Therefore, this study aimed to determine a cut-off point for the PhA capable of discriminating HD patients with reduced exercise tolerance.

## Methods

### Ethical aspects

It is characterized as a cross-sectional study. The reporting of this study followed the guideline of the STROBE statement [20]. In order to investigate the objective of this cross-sectional study, a secondary analysis of data from the initial evaluation of an ongoing randomized, double-blind clinical trial was performed [21]. This randomized, double-blind clinical trial was approved by the Human Research Ethics Committee of Hospital Sírio-Libanês (approval protocol no. 2017–95) and was registered in the Clinical Trials database (no. NCT03779126, date of first registration 19/12/2018). All patients signed the informed consent form.

### Patients

The sampling of the study was by convenience, being recruited patients from the Nephrology and Dialysis Center of Hospital Sírio-Libanês, São Paulo, São Paulo - Brazil. The inclusion criteria were: (1) patients with CKD undergoing HD; (2) older than 18 years; (3) without a pacemaker or other non-removable metallic device; (4) without cognitive or motor deficit that would limit the performance of the evaluations; and (5) without regular physical activity practice (more than twice a week). The exclusion criteria for this study were: (1) inability to perform assessments within technical acceptability criteria; and (2) cardiorespiratory instability (intolerant dyspnea, angina, pallor, diaphoresis or syncope) during the tests.

### Outcome measures

The evaluations were performed on three different days (Monday, Wednesday and Friday, or Tuesday, Thursday and Saturday), before the HD session. On the first day, the patients underwent anthropometry, inflammatory and nutritional status, and peripheral muscle strength assessment, using the Medical Research Council (MRC) scale and handgrip strength (HGS). In addition, venous blood was collected for analysis of biochemical markers. On the second day, patients underwent body composition and exercise tolerance assessment. Finally, on the third day, patients underwent lower limb muscle strength assessment, using isokinetic dynamometry.

Anthropometric assessment: body mass was assessed using a previously calibrated digital scale (Personal; Filizola, São Paulo, Brazil), while the height was measured using a stadiometer (Personal; Filizola, São Paulo, Brazil). With the value of the body mass index (BMI), the patients were classified as underweight (< 18.5 kg/m^2^), eutrophic (18.5–24.99 kg/m^2^), overweight (25–29.99 kg/m^2^) or obese (≥ 30 kg/m^2^) [22].

Inflammatory and nutritional status assessment: the Malnutrition and Inflammation Score (MIS) was used, which considers ten aspects: weight change after HD, dietary intake, gastrointestinal symptoms, functional capacity, comorbidities, fat reserve, muscle mass, BMI, albumin and ferritin. The score ranges from zero to 30, with higher scores representing worse inflammatory and nutritional status [23, 24].

MRC scale assessment: six muscle groups (shoulder abductors, elbow flexors, wrist extensors, hip flexors, knee extensors, and dorsiflexors) were bilaterally evaluated and scored from zero to five according to force production. The maximum value on this scale is 60 points and reflects greater peripheral muscle strength [25].

HGS assessment: was performed according to the American Society of Hand Therapists [26], using a hydraulic dynamometer (SH 5001; SAEHAN corporation, Yangdeok-Dong, South Korea). The evaluation of the dominant upper limb was prioritized, except in cases of arteriovenous fistula in that limb. Three measurements were performed, with an interval of one minute between them for rest. The highest value obtained was considered for analysis. The reference equation proposed by Novaes et al. [27] was used to calculate the predictive value.

Analysis of biochemical markers: blood collection through the HD access (catheter or arteriovenous fistula) was performed by the sector’s nursing team without needing a new puncture. Subsequently, the material was sent to a laboratory to analyze the following biochemical markers: creatinine, urea, lactate, ferritin, albumin, and insulin-like growth factor I (IGF-1).

Body composition assessment: the electrical bioimpedance (Body composition monitor; Fresenius Medical Care Renal Pharma Ltd., Wanchi, Hong Kong) was used, and the evaluation was performed with the patient in the supine position. Two self-adhesive electrodes adhered to the skin on the dorsal region of the hand, and two other electrodes adhered to the skin on the dorsal area of the ipsilateral foot. The skin of these regions was cleaned with a 70% alcohol swab before placing the electrodes. All metallic materials were removed from the patient’s proximity for evaluation [19]. The measured variables were: lean tissue index (LTI), fat tissue index (FTI) and PhA. All data obtained were analyzed by the software Fresenius Medical Care (Renal Pharma Ltd., Wanchi, Hong Kong), and the PhA was measured at a frequency of 50 kHz.

Exercise tolerance assessment: the six-minute step test (6MST) was used, a tool with clinimetric properties already established in the literature [28, 29]. To perform the test, a 15-cm step was positioned close to the wall to avoid displacement during the execution. Upper limb support was not allowed to perform the test. The patient was instructed to go up and down as many steps as possible in six minutes. The test could be interrupted if the patient presented any limiting symptoms without deactivating the timer. During the test execution, standardized incentive phrases were used every minute [30]. The parameters: blood pressure, heart rate, pulse oxygen saturation, respiratory rate, and subjective sensation of dyspnea and lower limb fatigue (assessed by the modified Borg scale [31]) were measured before the test, at the end, and after two minutes of recovery. The predictive value was calculated using the reference equation proposed by Arcuri et al. [32]. Based on previous studies, in which HD patients presented performance close to 50% of the normal values in the field tests [19, 33, 34], the reduced exercise tolerance was defined by performance less than or equal to 50% of the predicted value in the 6MST.

Lower limb muscle strength assessment: the isokinetic dynamometry (Biodex System 3; Biodex Medical Systems, Inc., New York, USA) was used to measure the peak torque of the knee extensor muscles of both lower limbs. For the evaluation, the patient was positioned sitting on the equipment with the back supported, and belts were used to stabilize the chest, pelvis, and thigh. Five maximum repetitions were performed at an angular velocity of 60°/s, using standardized phrases of encouragement [35]. The highest value obtained was considered for analysis.

### Sample size

The sample size calculation was performed using the SigmaPlot software version 11, and was based on the study by Kang et al. [36], in which a difference of 106 m was observed in the six-minute walk test and a standard deviation of 94 m in the patients with low PhA. Considering α = 0.05, a power of 80% and a sample loss of 10%, the total sample size was 31 patients.

### Statistical analysis

Data were analyzed using IBM SPSS (Statistical Package for Social Sciences) version 25. The Shapiro-Wilk test was used to assess the normality of the data, and they were represented according to their distribution on the Gauss curve (mean and standard deviation, or median and interquartile range). Parametric tests were used to analyze data with normal distribution and equivalent non-parametric tests were used to analyze data with non-normal distribution. Pearson’s coefficient evaluated the correlation between PhA and values (absolute and % of predicted) of 6MST. The correlations were classified as: low (0.26 to 0.49); moderate (0.50 to 0.69); high (0.70 to 0.89); and very high (0.90 to 1.00) [37]. A simple linear regression analysis was performed to investigate the influence of PhA on 6MST performance. The receiver operating characteristic curve (ROC curve) was applied and the area under the curve (AUC) value was analyzed to determine the ability of PhA to discriminate HD patients with reduced exercise tolerance. The AUC value was classified as: poor (0.60 ≤ AUC < 0.70); acceptable (0.70 ≤ AUC < 0.80); good (0.80 ≤ AUC < 0.90); and excellent (0.90 ≤ AUC) [38]. To determine the cut-off point, the highest sensitivity and specificity values ​​were considered. Finally, Fisher’s exact test, independent t-test, and Mann-Whitney U-test were used to compare data among patients with 6MST performance above and below 50% of the predicted value. For all analyses, the significance level adopted was *p* < 0.05.

## Results

**Fig. 1:**
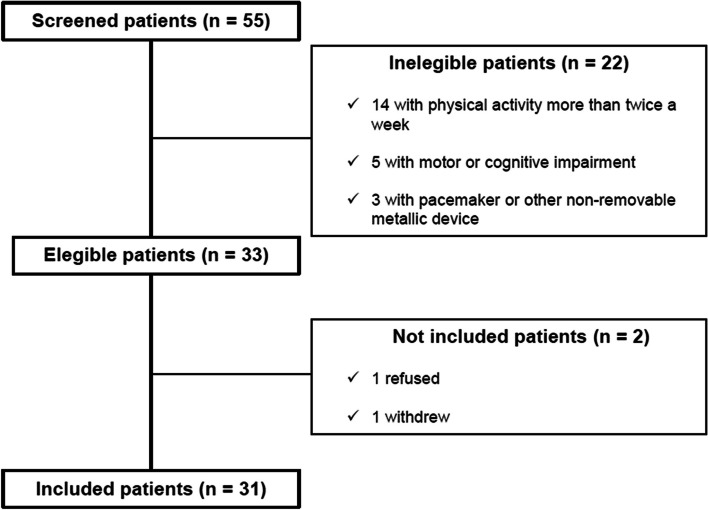
Study flowchart

Overall, 55 HD patients were screened, and 22 were considered ineligible for inclusion in the study. Thirty-three patients were eligible; however, one refused to participate in the study, and another withdrew (Fig. 1).

Our sample consisted of 31 individuals (80.6% men), with a median age of 69 years, a median time on HD of 23 months, and mean PhA of 4.0°. An average of 71% of the predicted value was observed for the HGS and 57.4 Nm and 65.1 Nm for the right and left lower limb peak torque, respectively. Regarding exercise tolerance, patients presented an average of 67.6 steps, corresponding to 50.5% of the predicted value in the 6MST. Fifteen patients (48.4%) presented performance less than or equal to 50% of the predicted value in the 6MST. Patients with reduced exercise tolerance had lower levels of creatinine, urea, albumin and IGF-1. They also had lower PhA, higher MIS, lower HGS, and lower peak torque in the right and left lower limbs when compared to patients with higher exercise tolerance (Table 1).

**Table 1:** Demographic and clinical characteristics of the patients

Variables	All patients (*n* = 31)	6MST ≤ 50% predicted (*n* = 15)	6MST > 50% predicted (*n* = 16)	*p*
Gender (M/F)	25 / 6	13 / 2	12 / 4	0.65
Age (years)	69.0 (54.0–83.0)	72.5 ± 10.8	61.5 ± 20.1	0.07
Body mass (Kg)	80.1 ± 14.6	81.0 (72.0–95.1)	76.7 (64.4–83.1)	0.12
Height (m)	1.7 ± 0.1	1.7 (1.7–1.8)	1.7 (1.6–1.7)	0.15
BMI (Kg/m^2^)	26.4 (25.1–30.2)	29.3 ± 5.7	26.4 ± 3.2	0.09
Time on HD (months)	23.0 (9.0–48.0)	23.0 (9.0–56.0)	24.0 (9.3–47.8)	0.78
**Biochemical markers**				
Creatinine (mg/dL)	8.7 ± 3.3	7.5 ± 2.0	9.9 ± 3.9	0.04*
Urea (mg/dL)	158.9 ± 31.8	144.0 ± 27.9	172.9 ± 29.4	0.009*
Kt/V	1.3 ± 0.4	1.2 ± 0.2	1.3 ± 0.4	0.54
Albumin (mg/dL)	4.0 ± 0.3	3.8 ± 0.3	4.1 ± 0.2	0.02*
Ferritin (mg/dL)	363.2 ± 182.7	321.9 ± 176.8	401.9 ± 185.2	0.23
Lactate (mg/dL)	9.9 ± 4.6	11.0 ± 4.6	8.9 ± 4.6	0.23
IGF-1 (mg/dL)	162.0 (131.0–191.0)	149.7 ± 29.8	194.3 ± 76.4	0.04*
**Body composition**				
LTI (Kg/m^2^)	10.2 (9.5–14.4)	9.7 (8.7–14.4)	10.5 (9.8–13.8)	0.33
FTI (Kg/m^2^)	15.0 ± 6.1	16.0 ± 7.8	14.1 ± 3.9	0.39
PhA (°)	4.0 ± 1.1	3.3 ± 0.5	4.7 ± 1.0	< 0.001*
MIS (points)	6.4 ± 2.8	7.9 ± 2.9	5.1 ± 1.9	0.003*
**Peripheral muscle strength**				
MRC (points)	52.0 (48.0–60.0)	48.0 (48.0–54.0)	55.0 (48.0–60.0)	0.07
HGS (Kgf)	26.0 (20.0–34.0)	22.8 ± 6.7	30.4 ± 8.2	< 0.001*
HGS (% predicted)	71.0 ± 22.9	59.2 (44.7–73.8)	74.7 (64.9–91.1)	0.04*
Peak torque RLL (Nm)	57.4 ± 18.3	46.3 ± 15.8	67.8 ± 14.1	< 0.001*
Peak torque LLL (Nm)	65.1 ± 22.7	54.2 ± 18.7	75.3 ± 21.8	0.007*
**Exercise tolerance**				
6MST (steps)	67.6 ± 41.0	34.7 ± 21.7	98.4 ± 28.8	< 0.001*
6MST (% predicted)	50.5 ± 28.5	27.5 ± 17.0	72.0 ± 18.3	< 0.001*

*Abbreviations: 6MST* six-minute step test, *M* male, *F* female, *BMI* body mass index, *HD* hemodialysis, *IGF-1* insulin-like growth factor I, *LTI* lean tissue index, *FTI* fat tissue index, *PhA* phase angle, *MIS* Malnutrition and Inflammation Score, *MRC* Medical Research Council, *HGS* handgrip strength, *RLL* right lower limb, *LLL* left lower limb.  *p < 0.05 indicates a significant difference compared to patients with 6MST ≤ 50% predicted

**Fig. 2:**
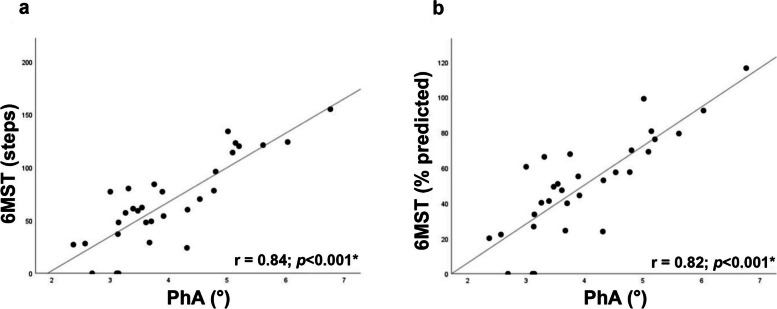
Correlation between PhA and absolute value (**a**) and % predicted in the 6MST (**b**). Abbreviations: PhA, phase angle; 6MST, six-minute step test. *p < 0.05

The Fig. 2 illustrates the high correlations observed between PhA and absolute value (r = 0.84; *p* < 0.001) and % predicted in the 6MST (r = 0.82; *p* < 0.001). In the simple linear regression model, the PhA could predict 68% of the performance in the 6MST (Table 2). This way, it was possible to determine the following predictive equation:

$$6MST\;(\%\;predicted)\;=\;(22.14\;\times\;PhA)\;-\;38.23$$

**Table 2:** Simple linear regression with predictor model for 6MST performance (% predicted)

Linear regression	Coefficient of regression	SE	95% CI	R^2^	*p*
Constant	−38.23	11.71	−62.17 to −14.29	–	0.003*
PhA (°)	22.14	2.83	16.36 to 27.92	0.68	< 0.001*

*Abbreviations: 6MST* six-minute step test, *SE* standard error, *95% CI* 95% confidence interval. *p < 0.05

**Fig. 3:**
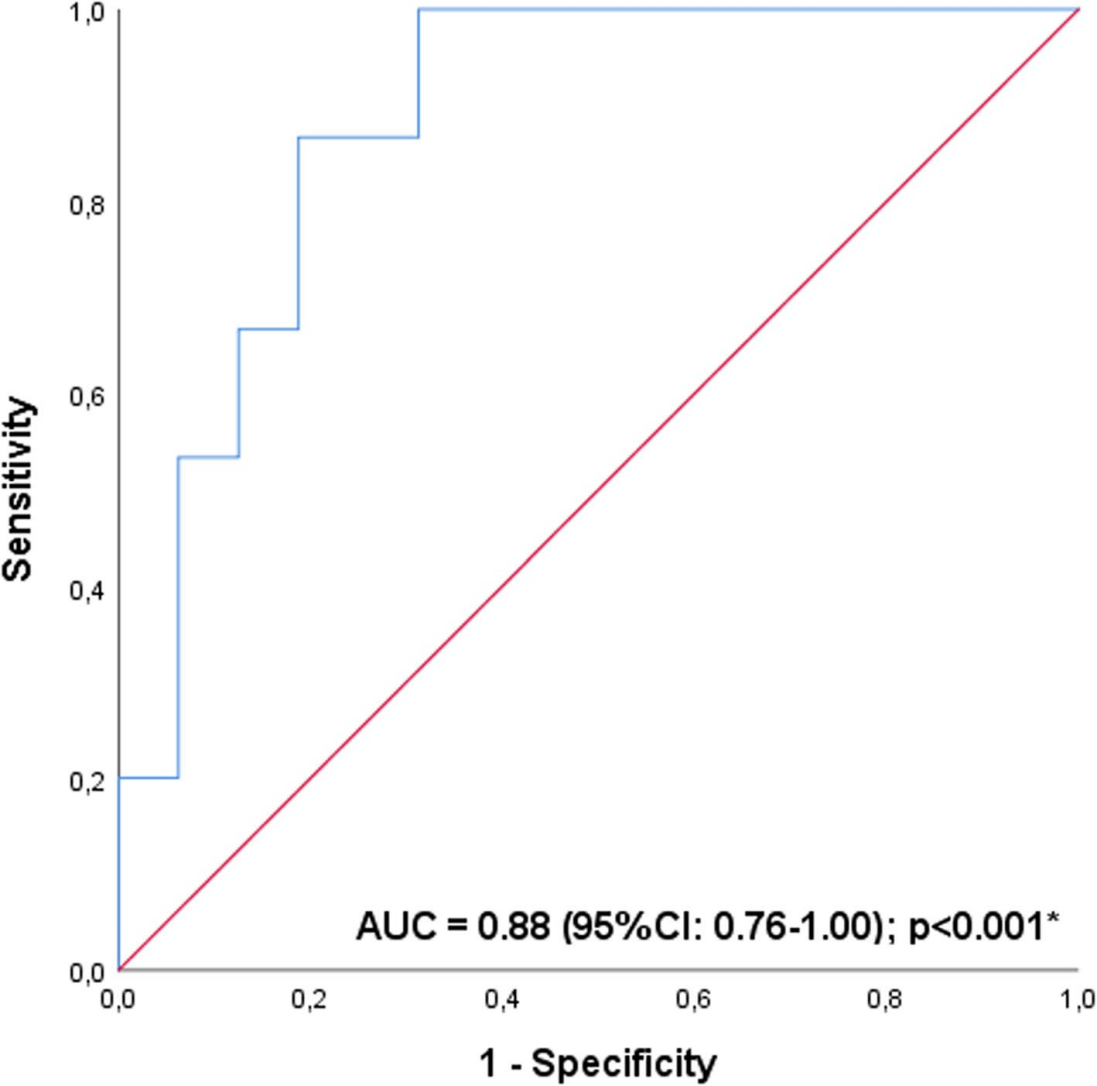
ROC curve for the cut-off point of the PhA capable of discriminating HD patients with reduced exercise tolerance. Abbreviations: ROC curve, receiver operating characteristic curve; PhA, phase angle; AUC, area under the curve; 95%CI, 95% confidence interval. *p < 0.05

The ROC curve indicated a cut-off point of 3.73° for the PhA (sensitivity = 87%, specificity = 81%, and AUC = 0.88 [95% CI: 0.76–1.00]; p < 0.001) capable of discriminate HD patients with reduced exercise tolerance (Fig. 3).

## Discussion

This study’s main finding was to determine a cut-off point of 3.73° for the PhA, based on the sensitivity and specificity values, to distinguish HD patients with reduced exercise tolerance. Corroborating our result, previous studies observed that lower PhA values were associated with increased protein energy wasting, lower rectus femoris muscle thickness, lower functional performance (peripheral muscle strength, gait speed and distance covered in the six-minute walk test) and increase of frailty in HD patients [15, 36, 39, 40]. In these studies, patients with lower functional performance had average PhA between 3.00° and 3.89°, values ​​similar to the cut-off point observed in our study. Another interesting result observed in our sample was that PhA explained 68% of the performance variability in 6MST. Studies indicate that PhA reflects the integrity of the cell membrane, the number of cells, and the performance of their functions [11, 13, 14]. Thus, as the skeletal muscle system is one of the gears required during the exercise [41, 42], the proper functioning of this system will reflect in better performance in the exercise tolerance test. Furthermore, as the 6MST is a tool that demands greater metabolic work, because it requires the lower limbs to work against gravity [43], it was expected that the PhA would influence the patients’ performance in this assessment. Given these results, the PhA appears as a useful marker for indirectly monitoring exercise tolerance in this population.

Our sample showed an average performance of 50.5% of the predicted value in the 6MST, indicating reduced exercise tolerance. Exercise intolerance in patients with CKD can be explained by changes in the respiratory, cardiovascular, and muscular systems. For example, the presence of pulmonary congestion and ventilatory disorders may favor the onset of dyspnea and lead to exercise limitation [4, 5, 44]. On the other hand, muscle dysfunction, which occurs through several mechanisms in CKD and that results in reduced capillary density, lower oxidative capacity and lower force production, may also contribute to exercise intolerance [1, 45–48]. In this sense, this reduced exercise tolerance can result in lower levels of functionality and increased sedentary lifestyle, increasing the risk of death in this population. Confirming this rationale, previous studies observed an association between lower levels of physical activity and the risk of mortality in patients with CKD [7–9]. This reinforces the need for periodic monitoring of exercise tolerance in this population, aiming at referral to rehabilitation programs. For this reason, and based on our results, the PhA could be used to facilitate the screening of these patients.

The observed results were already expected when comparing the groups of patients with higher and lower exercise tolerance. Patients with performance ≤50% of the predicted value on the 6MST had lower levels of creatinine, urea and albumin, which are variables that can be affected by inflammation and worse nutritional status [49–51]. Higher MIS was also observed in these patients, which supports this rationale. In addition, this group also showed a reduction in muscle function markers (IGF-1, PhA, HGS, and peak torque), which indicates a more significant impairment of peripheral muscles and justifies the worse performance in the 6MST. Muscle dysfunction in CKD has a multifactorial etiology and results in increased catabolism and reduced protein synthesis [1, 2]. Furthermore, physical inactivity can accentuate this process and contribute to a more significant loss of muscle mass and strength [45]. For this reason, therapeutic modalities (neuromuscular electrical stimulation and resistance training) aimed at improving muscle function can favor the increase in exercise tolerance and should be prescribed for this population [52, 53].

This study has some limitations. First, hydration level is a variable that can influence the measurement of PhA [14]; however, all included patients were evaluated in the same condition (before the second HD session of the week). Another limitation was the use of a cut-off point not validated in the literature to define reduced exercise tolerance. However, the cut-off point of 50% of the predicted value was used, because previous studies reported that HD patients presented functional performance close to 50% of the normal values [19, 33, 34]. Furthermore, this cut-off point was similar to the mean and median values (50.5 and 50.9%, respectively) observed in our sample. It is suggested that further studies be carried out in order to investigate the ideal cut-off point to characterize exercise intolerance in this population. Another limitation is the restricted sample size with a predominance of male individuals, which may contribute to higher PhA values. Therefore, it is suggested that future investigations use larger samples with a more homogeneous distribution between men and women to confirm the cut-off point established in our study. Finally, our sample consisted of HD patients who did not practice regular physical activity (≤2 times a week), making it difficult to extrapolate the results to physically active patients. However, the study’s objective was to establish a cut-off point for PhA that would facilitate the identification of impairment exercise tolerance. Thus, patients who would benefit from this cut-off point were included in the study.

To our knowledge, our study pioneered investigating PhA as a predictor of reduced exercise tolerance in HD patients. In this way, the suggested cut-off point can facilitate the screening and referral of these individuals to rehabilitation programs.

## Conclusion

The cut-off point of 3.73° for the PhA is sensitive and specific to discriminate HD patients with reduced exercise tolerance. In these patients, worse inflammatory and nutritional status, lower PhA and greater impairment of peripheral muscle strength were observed.

### Supplementary Information

**Additional file 1.** STROBE Statement - Checklist of items that should be included in reports of cross-sectional studies.

## Data Availability

The datasets used and/or analyzed during the current study available from the corresponding author on reasonable request.
