# Supplementary material for: The phase angle cut-off point capable of discriminating hemodialysis patients with reduced exercise tolerance: a cross-sectional study
Source: BMC Sports Sci Med Rehabil. 2024 Feb 2;16:34. doi: 10.1186/s13102-024-00825-5 (PMC10835815; doi:10.1186/s13102-024-00825-5)
Supplement: Supplementary file 1 — Additional file 1. STROBE Statement - Checklist of items that should be included in reports of cross-sectional studies. [file 13102_2024_825_MOESM1_ESM.docx]

Additional file 1. STROBE Statement - Checklist of items that should be included in reports of cross-sectional studies.

|  | **Item** | **Recommendation** | **Section** | **Paragraph/subheading** |
| --- | --- | --- | --- | --- |
| **Title and abstract** | 1 | a) Indicate the study’s design with a commonly used term in the title or the abstract | Title | Title |
|  |  | b) Provide in the abstract an informative and balanced summary of what was done  and what was found | Abstract | Methods and Results |
| **Introduction** | | | | |
| Background | 2 | Explain the scientific background and rationale for the investigation being reported | Background | Paragraphs 1-3 |
| Objectives | 3 | State specific objectives, including any prespecified hypotheses | Background | Paragraph 3 |
| **Methods** | | | | |
| Study design | 4 | Present key elements of study design early in the paper | Methods | Ethical aspects |
| Setting | 5 | Describe the setting, locations, and relevant dates, including periods of recruitment,  exposure, follow-up, and data collection | Methods | Patients |
| Participants | 6 | Give the eligibility criteria, and the sources and methods of selection of  participants | Methods | Patients |
| Variables | 7 | Clearly define all outcomes, exposures, predictors, potential confounders, and effect  modifiers. Give diagnostic criteria, if applicable | Methods | Outcome measures |
| Measurement | 8 | For each variable of interest, give sources of data and details of methods of  assessment (measurement). Describe comparability of assessment methods if there is  more than one group | Methods | Outcome measures |
| Bias | 9 | Describe any efforts to address potential sources of bias | Methods | Outcome measures |
| Study size | 10 | Explain how the study size was arrived at | Methods | Sample size |
| Quantitative variables | 11 | Explain how quantitative variables were handled in the analyses. If applicable,  describe which groupings were chosen and why | Methods | Statistical analysis |
| Statistical methods | 12 | a) Describe all statistical methods, including those used to control for confounding | Methods | Statistical analysis |
|  |  | b) Describe any methods used to examine subgroups and interactions | Methods | Statistical analysis |
|  |  | c) Explain how missing data were addressed | Not applicable | Not  applicable |
|  |  | d) ) If applicable, describe analytical methods taking account of sampling strategy | Not applicable | Not  applicable |
|  |  | e) Describe any sensitivity analyses | Methods | Statistical analysis |
| **Results** | | | | |
| Participants | 13 | a) Report numbers of individuals at each stage of study—eg numbers potentially  eligible, examined for eligibility, confirmed eligible, included in the study,  completing follow-up, and analysed | Results | Paragraph 1 and Fig.1 |
|  |  | b) Give reasons for non-participation at each stage | Results | Fig. 1 |
|  |  | c) Consider use of a flow diagram | Results | Fig. 1 |
| Descriptive data | 14 | a) Give characteristics of study participants (eg demographic, clinical, social) and  information on exposures and potential confounders | Results | Paragraph 2  and Table 1 |
|  |  | b) Indicate number of participants with missing data for each variable of interest | Not applicable | Not  applicable |
| Outcome data | 15 | Report numbers of outcome events or summary measures | Results | Paragraphs 2-4  and Table 1 |
| Main results | 16 | a) Give unadjusted estimates and, if applicable, confounder-adjusted estimates and  their precision (eg, 95% confidence interval). Make clear which confounders were  adjusted for and why they were included | Results | Paragraph 4 |
|  |  | b) Report category boundaries when continuous variables were categorized | Results | Table 1 |
|  |  | c) If relevant, consider translating estimates of relative risk into absolute risk for a  meaningful time period | Not applicable | Not  applicable |
| Other analyses | 17 | Report other analyses done—eg analyses of subgroups and interactions, and  sensitivity analyses | Results | Paragraphs 2-4  and Table 1 |
| **Discussion** | | | | |
| Key results | 18 | Summarise key results with reference to study objectives | Discussion | Paragraphs 1-3 |
| Limitations | 19 | Discuss limitations of the study, taking into account sources of potential bias or  imprecision. Discuss both direction and magnitude of any potential bias | Discussion | Paragraph 4 |
| Interpretation | 20 | Give a cautious overall interpretation of results considering objectives, limitations,  multiplicity of analyses, results from similar studies, and other relevant evidence | Discussion  Conclusion | Paragraph 5 |
| Generalisability | 21 | Discuss the generalisability (external validity) of the study results | Discussion  Conclusion | Paragraphs 4-5 |
| **Other information** | | | | |
| Funding | 22 | Give the source of funding and the role of the funders for the present study and, if  applicable, for the original study on which the present article is based | Funding |  |
